# The Influence of Different Light Angles During Standardized Patient Photographic Assessment on the Aesthetic Perception of the Face

**DOI:** 10.1007/s00266-021-02314-3

**Published:** 2021-05-13

**Authors:** Claudia A. Hernandez, John Mario Espinal, David Uribe Zapata, Daniel Coimbra, Michael Alfertshofer, Konstantin Frank, Jeremy B Green, Kristina Davidovic, Diana L. Gavril, Sebastian Cotofana

**Affiliations:** 1 CH Dermatologia, Medellin, Colombia; 2 Independent Photographer, Medellin, Colombia; 3 Department of Cosmetic Dermatology at Santa Casa de Misericórdia, Rio de Janeiro, Brazil; 4 Department for Hand, Plastic and Aesthetic Surgery, Ludwig – Maximilian University, Munich, Germany; 5 Skin Associates of South Florida, Skin Research Institute, Coral Gables, FL USA; 6 Department of Radiology and Medical School, University of Belgrade, Belgrade, Serbia; 7 Private Practice, Cluj-Napoca, Romania; 8 Department of Clinical Anatomy, Mayo Clinic College of Medicine and Science, Mayo Clinic, Stabile Building 9-38, 200 First Street, Rochester, MN 55905 USA

**Keywords:** Light, Aesthetic perception, Facial aging, Standardized photography

## Abstract

**Background:**

2D baseline and follow-up clinical images are potentially subject to inconsistency due to alteration of imaging parameters. However, no study to date has attempted to quantify the magnitude by which such images can be influenced.

**Objective:**

The objective of the present study is to identify the magnitude by which images can be influenced by changing the imaging light angle.

**Methods:**

This study is based on the evaluation of 2D frontal images of the face and included a total of 51 subjects of which *n* = 14 were males and *n* = 37 were females. Faces were photographed at 0°, 30°, and 60° light angle under identical and standardized conditions. Images were randomized and rated by 27 blinded raters for age, facial attractiveness, body mass index (BMI), temporal hollowing, lower cheek fullness, nasolabial sulcus severity, and jawline contour.

**Results:**

Facial attractiveness decreased, facial unattractiveness increased and the evaluated BMI (based on facial assessment) increased statistically significantly at 60°. The assessment of regional facial scores, i.e., temporal hollowing, lower cheek fullness, and jawline contour, showed no statistically meaningful changes both at 30° and at 60° light angle.

**Conclusion:**

The results indicate that there might be an observed blind range in light angle (0°–30°) which does not influence facial assessment. Increasing the light angle past the threshold value to 60° might result in a statistically significant impact on facial perception which should be accounted for when documenting and/or presenting facial 2D images.

**Level of Evidence V:**

This journal requires that authors assign a level of evidence to each article. For a full description of these Evidence-Based Medicine ratings, please refer to the Table of Contents or the online Instructions to Authors www.springer.com/00266.

## Introduction

Consistent pre- and post-procedure photographs are absolutely essential for aesthetic medicine [1–5]. Whether the intervention involves surgery, minimally invasive injectables, or energy-based devices, high quality before and after images are crucial for both patient and practitioner assessment of outcomes, and potentially for medicolegal considerations. Those who have practiced aesthetic medicine long enough have undoubtedly encountered the unhappy patient despite a successful outcome. The greatest frustration in this scenario is when the after images look worse than the before due to inconsistent image capture. Though this phenomenon is unfortunately known to busy aesthetic practitioners, the impact of inconsistent photography especially in regard to lighting variability has, to the authors’ knowledge, never been assessed in an objective fashion.

The inverse of this scenario is unfortunately all too common on social media channels, where the before image is clearly captured with a different environment to make the patient appear worse, and the after image with more flattering conditions (i.e., alteration in lighting, application of a filter, etc.) can create the false impression of a successful aesthetic intervention. In addition, a recent study has shown that “selfie” images in social media can have “*deleterious effects … on human mankind and well-being*” due to the increase in social anxiety, the decrease of confidence and the feeling of decrease in physical attractiveness [6]. This can create a bias toward an exaggerated outcome and can overestimate the aesthetic results which could obscure the effectiveness of a specific intervention and lead to disappointment in patients.

The objective of the present study is to identify the magnitude by which clinical images can be influenced by alterations of imaging parameters. To facilitate this investigation it was decided to keep all imaging parameters constant and to alter only one factor: angle of light. It was additionally decided to use facial images for the image assessment and to separate image capture from image assessment and from data analysis. With this study design, the investigators hoped to achieve the most objective analysis of what is typically a subjective assessment.

## Material and Methods

### Study Sample

This study is based on the evaluation of patient images and included a total of 51 subjects of which *n *= 14 were males and *n* = 37 were females. The mean age of the total sample was 33.5 (8.5) years of which *n* = 34 had a body mass index (BMI) of < 25 kg/m^2^, *n* = 13 had a BMI between 25 and 30 kg/m^2^, and *n *= 4 had a BMI of > 30 kg/m^2^.

Study participants were recruited and photographed at the dermatologic practice REDACTED and provided written informed consent for the use of both their personal and imaging data prior to their initiation into the study. The study was approved by the ethics committee of the REDACTED under the approval number: 9H16D88MB0350/2020. The study was conducted in accordance with regional laws (REDACTED) and good clinical practice between November and December 2020.

### Imaging Procedure

**Fig. 1:**
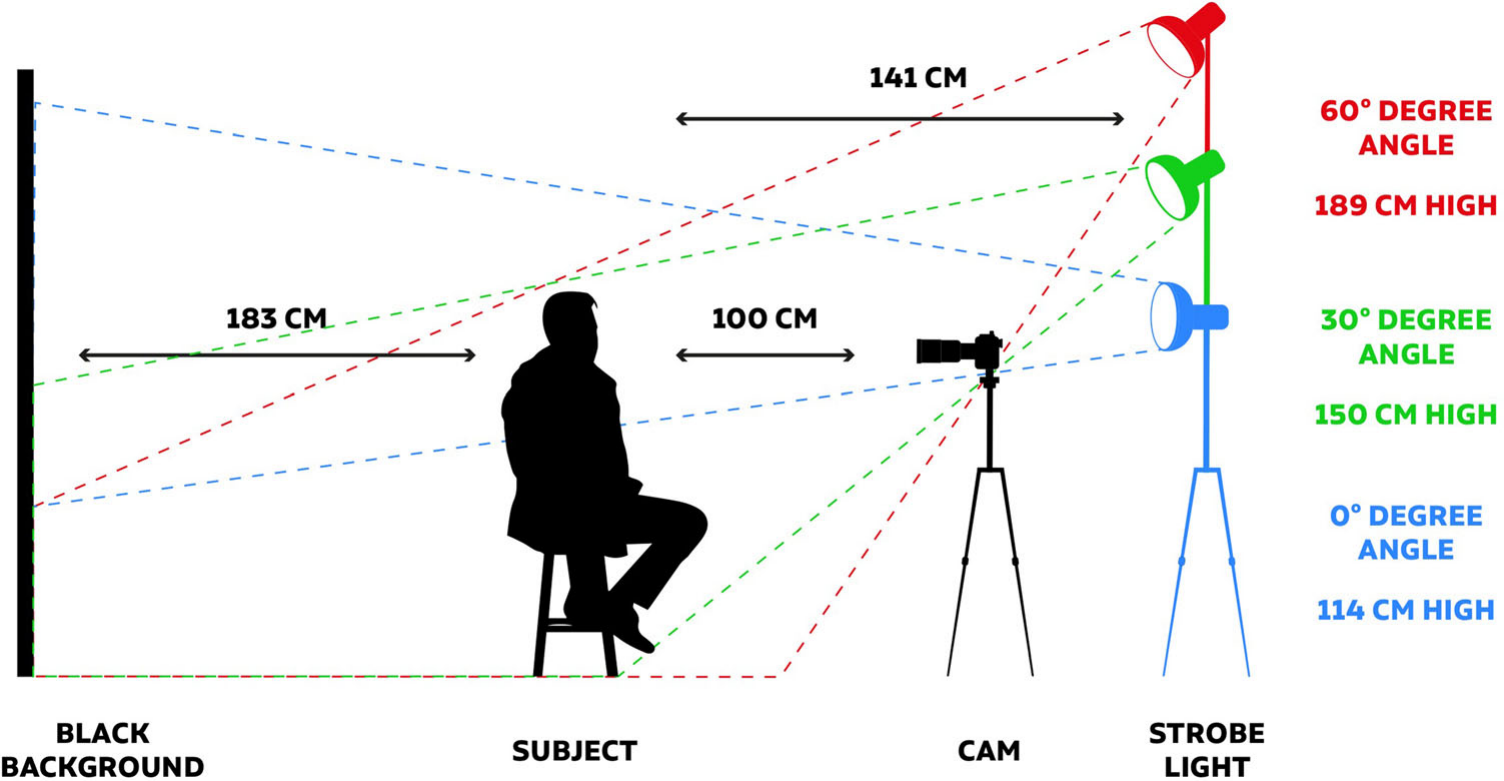
Graphic illustration of the image capture setup providing detailed measurements for reproducibility purposes.

All 51 study participants were photographed under standardized conditions in the same location, with the same photographic equipment and by the same person (J.M.E.) to ensure consistency during the imaging process. The following imaging parameters were documented: camera (Nikon D850, Nikon, Tokyo, Japan), lens (24–120 mm, Nikon, Tokyo, Japan), light source (strobe light Godox QS 400, Godox, Shenzhen, China), temperature of light (5600 °K), lens distance (120 mm), aperture (14), speed (1/160), and light exposure (ISO 64). Detailed measurements for patient and camera positioning are shown in Fig. 1.

**Fig. 2:**
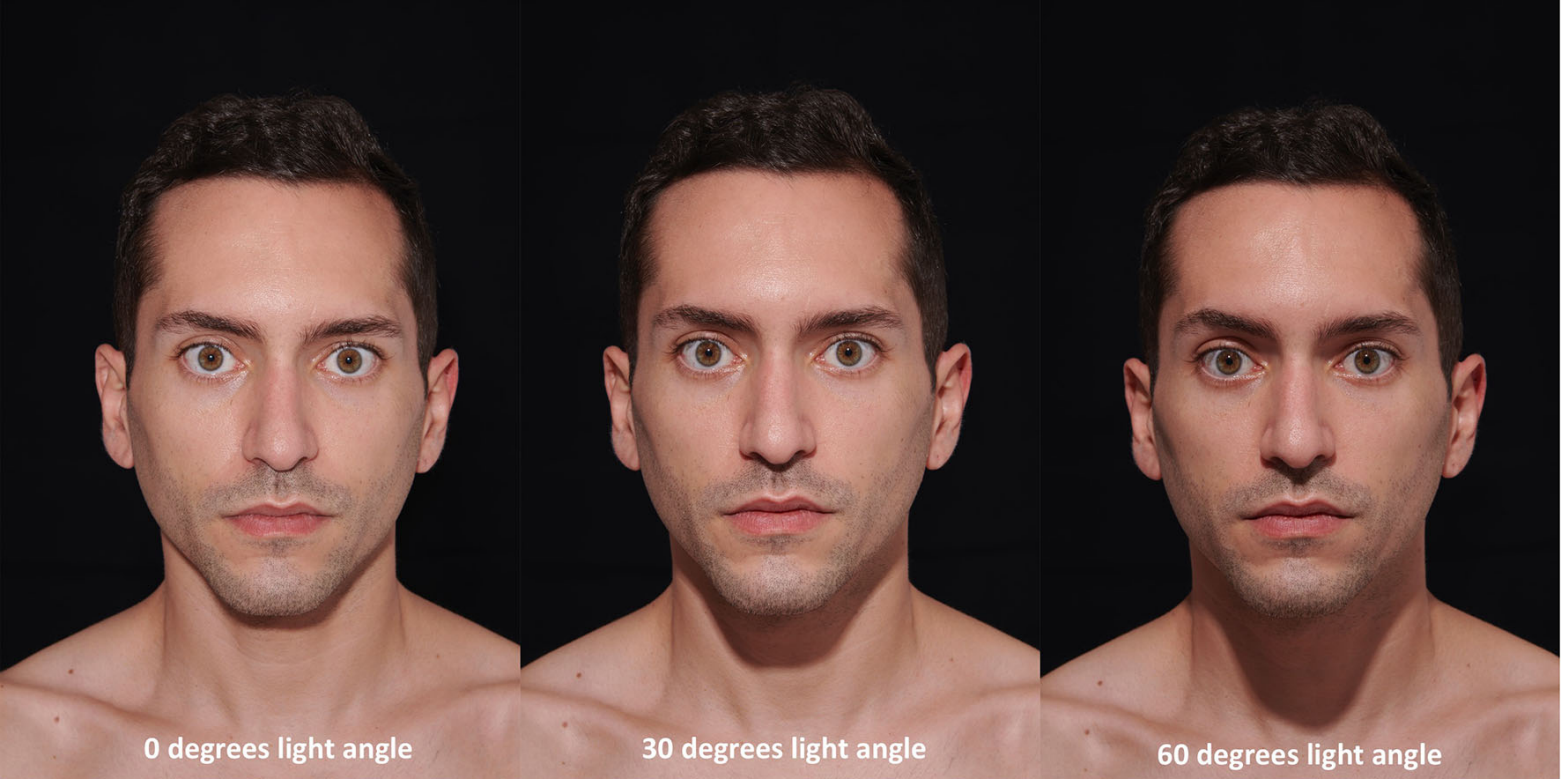
Male, study participant shown at 0°, 30°, and 60° light angle.

**Fig. 3:**
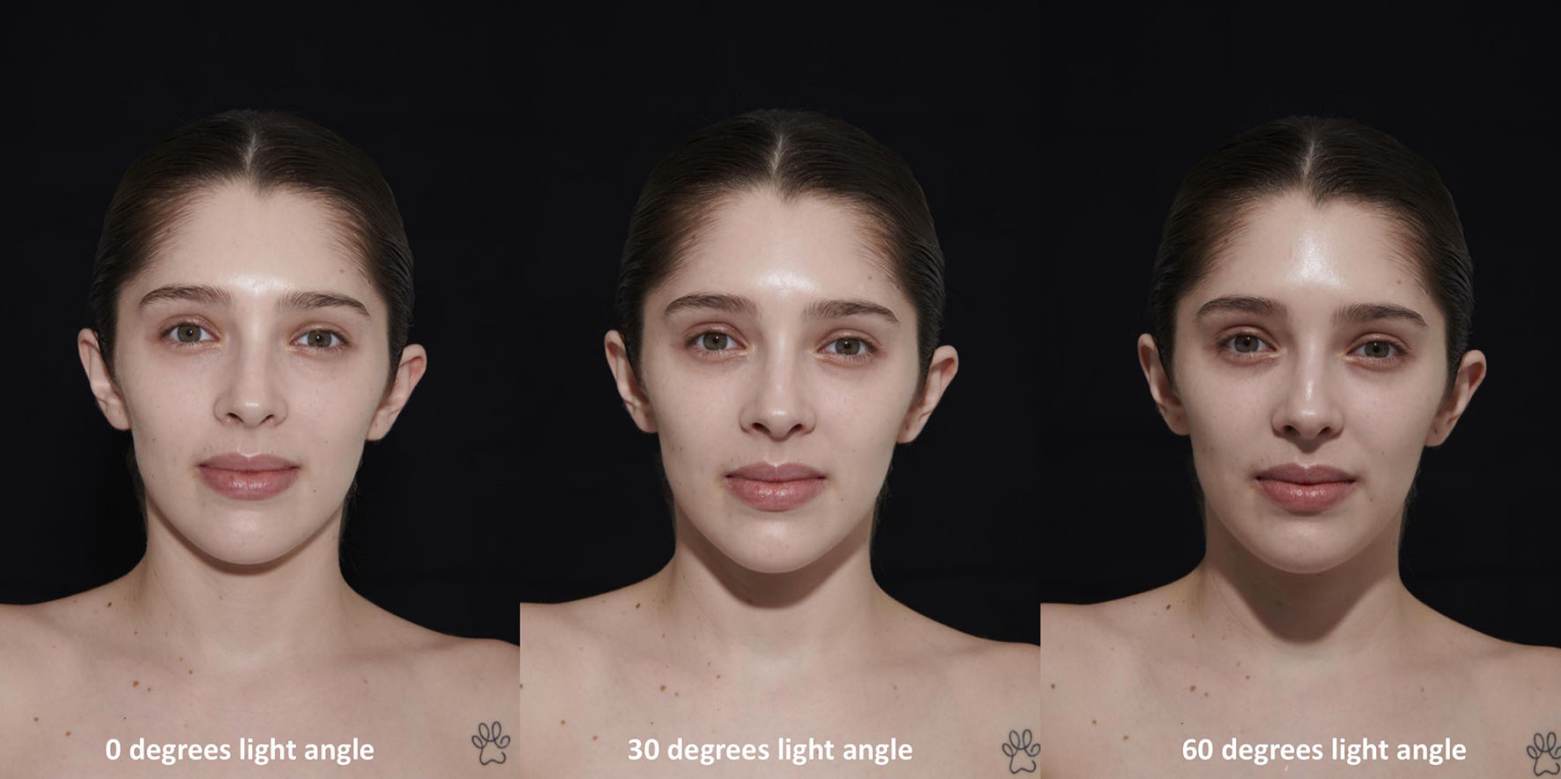
Female study participant shown at 0°, 30°, and 60° light angle.

Each of the 51 study participants was photographed at a light angle of 0°, 30°, and 60  resulting in set of three images per study participant. The three different light angles were obtained by increasing the height of the light source from 114 (= 0° light angle) to 150 cm (= 30  light angle) and finally to 189 cm (= 60° light angle). Before each image capture the above-mentioned light parameters were controlled and readjusted if needed to ensure consistency throughout the imaging process (Figs. 2, 3).

### Image Analysis

The obtained images were collected and digitally assessed for quality purposes. In a next step, the 153 images were randomized and re-arranged in a separate file for further evaluation. The randomized file was then sent out to 27 independent and blinded raters from six different countries (Serbia, Germany, the USA, Brazil, Romania, and Philippines) who had no exposure to or involvement with the image capture, the randomization process and were previously not informed about the background (different light angles) of the randomized patient images.

The 27 raters had varying medical backgrounds including plastic surgeons, dermatologists, radiologists, general medicine, and doctoral students in medicine. The independent and blinded raters were asked to assess the 153 randomized images for the following parameters:

- Age of the person shown in the image
- BMI of the person shown in the image (< 25 kg/m^2^, 25–30 kg/m^2^, > 30 kg/m^2^)
- Facial attractiveness rated on a five-point Likert scale: “I perceive the evaluated face as very attractive”: strongly disagree, disagree, neutral, agree, and strongly agree
- Facial unattractiveness rated on a five-point Likert scale: “I perceive the evaluated face as very unattractive”: strongly disagree, disagree, neutral, agree, and strongly agree
- Temporal hollowing scale rated on a five-point Likert scale: no (temporal hollowing), mild, moderate, severe, and very severe
- Lower cheek fullness scale rated on a five-point Likert scale: no (cheek volume loss), mild, moderate, severe, and very severe
- Nasolabial fold severity rated on a five-point Likert scale: no (nasolabial fold), mild, moderate, severe, and very severe
- Jawline contour scale rated on a five-point Likert scale: no (jawline contour loss), mild, moderate, severe, and very severe

### Analytic Procedure

Each of the 27 raters returned their completed rating sheets to the coordinating study center which was not involved in the process of image acquisition, picture randomization or image assessment.

To analyze the consistency (= reliability) across the assessment of independent raters, Cronbach’s alpha was calculated across the 27 raters’ response for each variable assessed.

Differences between the three different light angles were computed using generalized linear models with robust estimator utilizing linear (age) and ordinal logistic (all other variables) models.

Analyses were performed using SPSS Statistics 23 (IBM, Armonk, NY, USA) and differences were considered statistically significant at a probability level of ≤ 0.05 to guide conclusions. Values are presented as mean and standard deviation independent of their scale (linear or ordinal variables) for a better understanding of the readership.

## Results

### Assessment of Age

Cronbach’s alpha was for the assessment of age 0.981 across the 27 raters. The assessed mean age for 0° light angle was 35.68 (8.7) years, whereas for 30° it was 35.77 (8.8) years and for 60° it was 36.11 (8.9) years. Generalized linear models (linear) revealed that for an increase in 30° of light angle, study subjects were perceived to be 0.09 years older (*p *= 0.792) while an increase to a light angle of 60°, study subjects were perceived to be 0.44 years older (*p *= 0.192) (Table 1).

**Table 1:** Generalized linear models results presented as the beta value of each respective facial assessment in relation to the assessment at 0° light angle

	Beta at 30°	*p*-value	Beta at 60°	*p*-value
Age	0.09	0.792	0.44	0.192
BMI	0.19	0.009	0.23	0.002
Facial attractiveness	0.01	0.938	0.16	0.024
Facial unattractiveness	0.07	0.314	0.16	0.020
Temporal hollowing scale	0.01	0.942	0.03	0.649
Lower cheek fullness scale	− 0.01	0.845	− 0.01	0.862
Nasolabial fold severity	− 0.06	0.398	0.16	0.020
Jawline contour scale	− 0.07	0.338	0.03	0.709

At 30° light angle, age was perceived a mean of 0.09 years older when compared to the assessment at 0° light angle; this occurred with a probability value of 0.792.

### Assessment of Body Mass Index

**Fig. 4:**
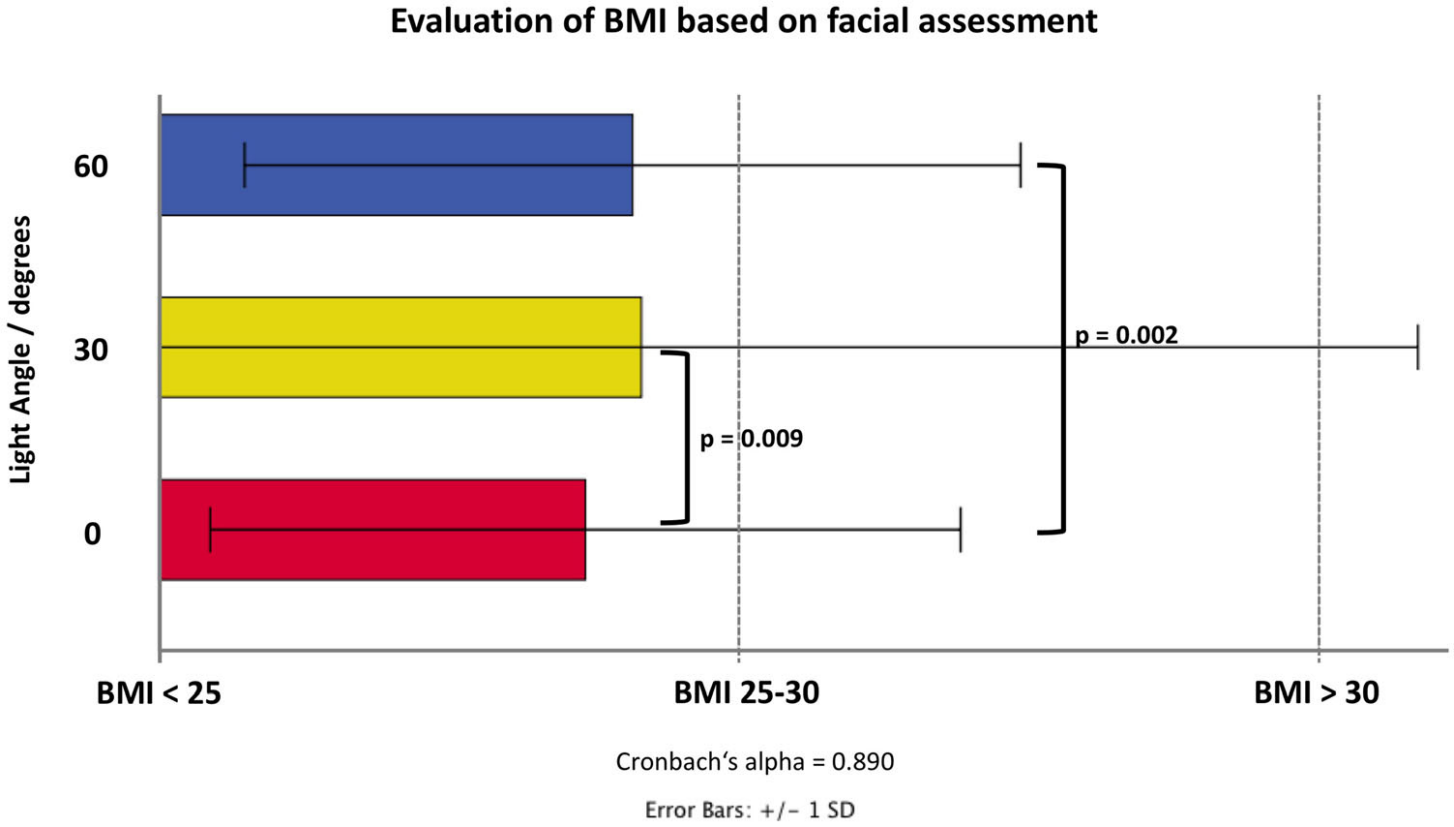
Bar graph showing the results of the evaluation of the (facial) body mass index (BMI) for the three different BMI groups at each of the investigated light angles: 0°, 30°, 60°.

The assessment of BMI had a reliability of 0.890 (Cronbach’s alpha) across the 27 raters, when asked to group the inspected study participants into the following three groups: 1: < 25 kg/m^2^; 2: 25–30 kg/m^2^; 3: > 30 kg/m^2^. The mean value for the group assessment was 1.74 (0.65) for 0° light angle, 1.83 (1.3) for the 30° light angle and 1.82 (0.67) for the 60° light angle. Generalized linear models (ordinal logistic) revealed a statistically significant increase in the BMI rating of the inspected images with a higher rating of 0.19 for the increase in 30° of light angle compared to 0° (*p *= 0.009) and with a higher rating of 0.23 for the increase of 60° of light angle (*p *= 0.002) (Fig. 4).

### Assessment of Facial Attractiveness

**Fig. 5:**
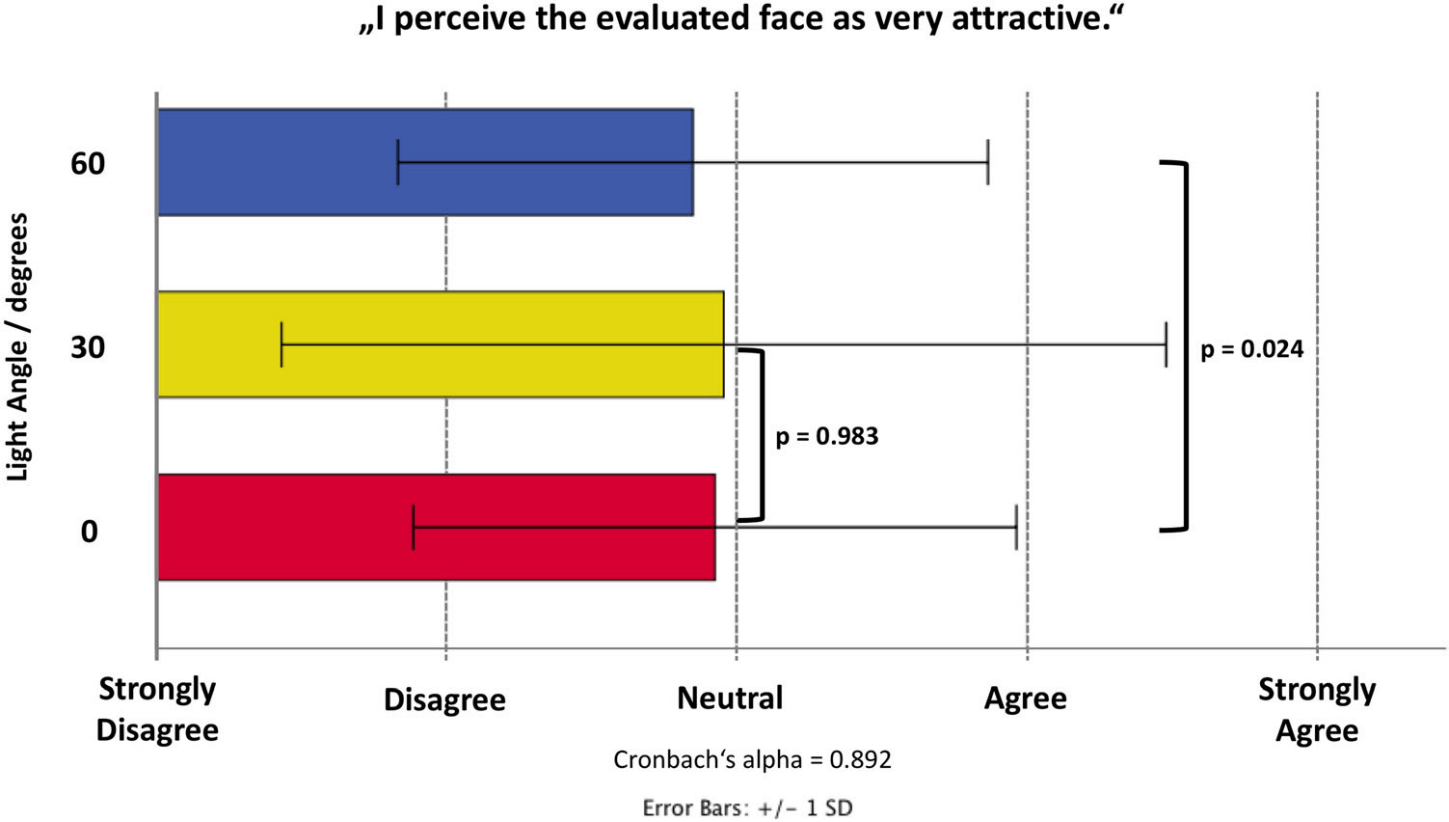
Bar graph showing the results of the evaluation of facial attractiveness as assessed on a five-point Likert scale at each of the investigated light angles: 0°, 30°, 60°.

Facial attractiveness was rated across the 27 raters with a reliability of 0.892 (Cronbach’s alpha). The assessed mean value (from a Likert scale ranging from 1 to 5, worst to best) for 0° light angle was 2.93 (1.0), whereas for 30° light angle it was 2.96 (1.5) and was for 60° light angle 2.85 (1.0). Generalized linear models (ordinal logistic) revealed that for the increase by 30° in light angle facial attractiveness was rated 0.01 points lower compared to 0° (*p *= 0.938) whereas the increase by 60° light angle resulted in the decrease in facial attractiveness by 0.16 points (*p *= 0.024) (Fig. 5).

### Assessment of Facial Unattractiveness

**Fig. 6:**
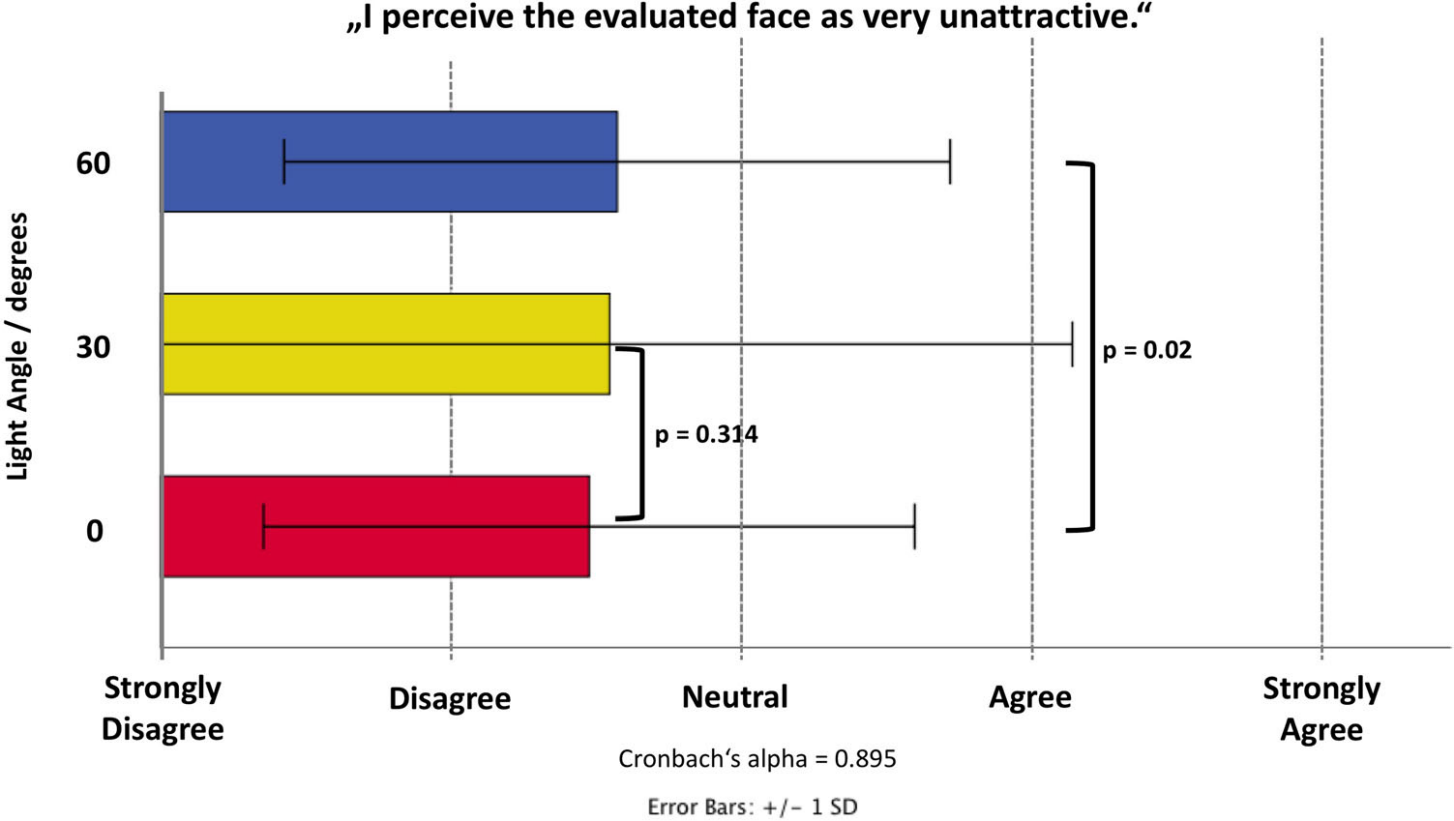
Bar graph showing the results of the evaluation of facial unattractiveness as assessed on a five-point Likert scale at each of the investigated light angles: 0°, 30°, 60°.

Reliability analysis across the 27 raters revealed a Cronbach’s alpha of 0.895 for the rating of facial unattractiveness. Raters were asked to strongly disagree (rated as 1 out of 5) or to strongly agree (rated as 5 out of 5) if they perceived the inspected images as unattractive. The mean value for the unattractiveness rating was 2.48 (1.1) for 0° light angle, 2.55 (1.6) for 30° light angle and 2.57 (1.1) for the 60° light angle. Generalized linear models (ordinal logistic) revealed that the increase in 30° of light angle resulted in greater facial unattractiveness by 0.07 points (*p *= 0.314) whereas increasing the light angle to 60° resulted in an increased unattractiveness of 0.16 points (*p *= 0.020) (Fig. 6).

### Assessment of Facial Scores

**Fig. 7:**
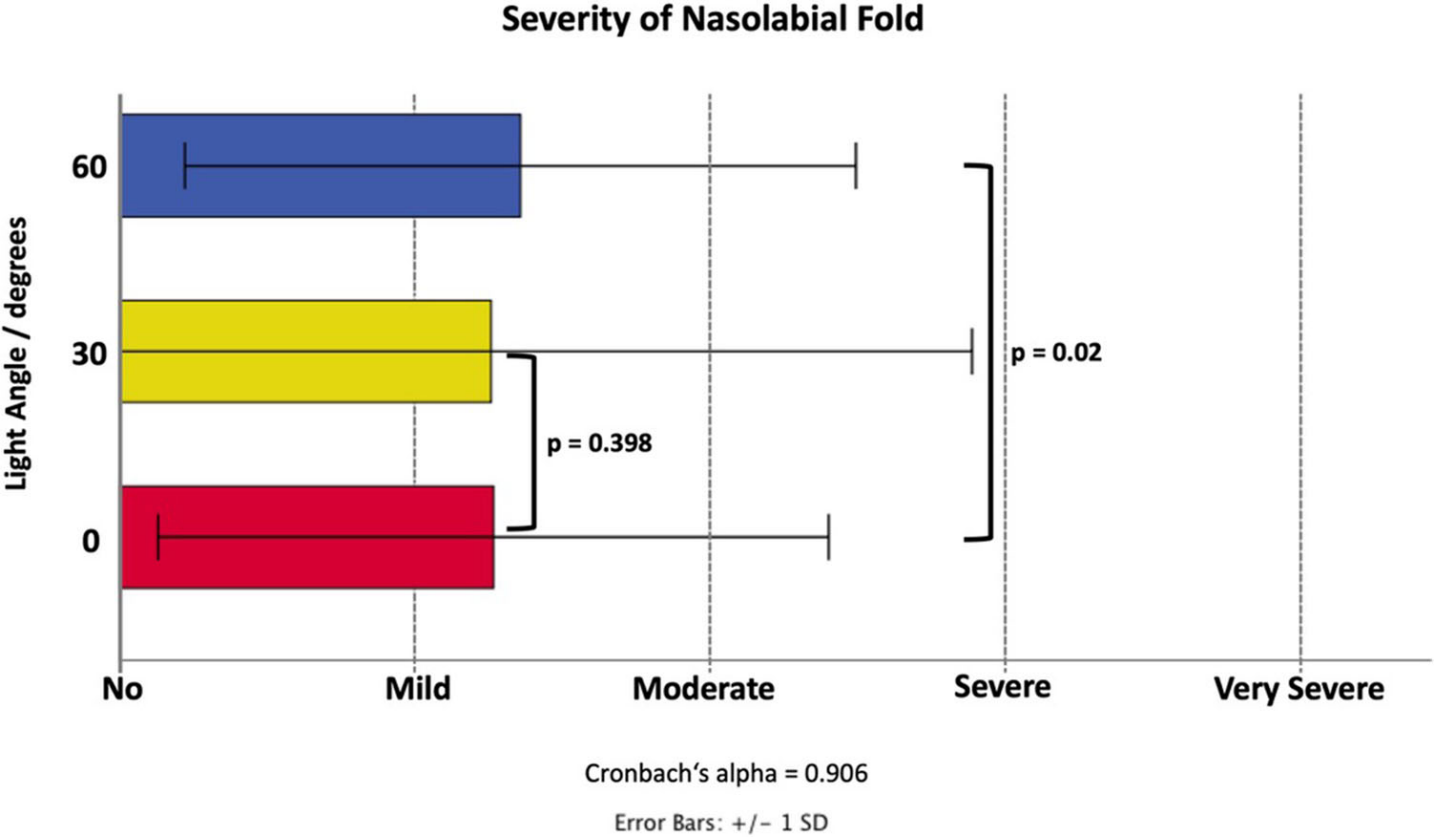
Bar graph showing the results of the evaluation of nasolabial fold severity as assessed on a 5-point Likert scale at each of the investigated light angles: 0°, 30°, 60°.

The reliability for the assessed facial scores was 0.607 for temporal hollowing, 0.628 for lower cheek fullness, was 0.859 for jawline contour, and 0.906 for nasolabial fold severity. None of the assessed scores revealed a statistically significant change under the influence of an increase in light angle except the severity of the nasolabial fold. Here, generalized linear models (ordinal logistic) revealed that the increase by 60° in light angle resulted in an increased severity rating (0–4, best to worst) of 0.159 points with *p *= 0.020 (Fig. 7).

## Discussion

Inconsistency in pre- and post-aesthetic intervention 2D photography can obscure outcome assessment and thus potentially patient and clinician procedural satisfaction. Perhaps more seriously, inconsistent photography could prove deleterious should any medicolegal considerations arise. It is well known that changing light angles affects the contours of the face and body [7]. For instance, when performing pre-treatment photography of cellulite dimples on the buttock and thighs, higher light angles (overhead light) accentuate the depth of the depressions and facilitate identification of more subtle surface irregularities that could be thus targeted with interventions [8]. Similarly, during minimally invasive facial soft tissue filler injections, overhead light accentuates contours of the face and facilitates identification of light and shadow prior to intervention. In contrast, lower light angles used in fashion photography seeks to avoid the creation of shadows (i.e., sites like infraorbital hollows) which could connote fatigue/advanced age [9].

The objective of this study was to attempt to quantify the influences of the change in light angle on the assessment of age, BMI, facial attractiveness and various facial assessment scores. The varying light angle (not camera angle) was obtained by altering the height of the light source from 114 (= 0° light angle) to 150 cm (= 30° light angle) and finally to 189 cm (= 60° light angle). In this way the facial illumination angle was changed which influenced the light and shadow proportions of the face.

A strength of the study is the blinded design which ensures objectivity of the data presented. Image capture and image randomization occurred separately from image assessment and separately from data analysis and manuscript conception. Image capture was performed in a standardized and controlled fashion by a professional photographer with more than 10 years of experience in professional and medical and fashion imaging. The captured images were inspected for quality control and randomized. After the randomization process the images were sent to the 27 international raters who were blinded to the scope of the study. It can be presumed that due to the repeated assessment of the same study subject, some raters may have identified the alternating light angles during their assessment. However, this process cannot be influenced a priori or accounted for during the image assessment. The 27 blinded raters were ethnically diverse and included Caucasian, Asian, and Latin-American physicians. To account for the heterogeneity of the 27 raters, each variable investigated was controlled for their Cronbach’s alpha; this measure of internal consistency exposes inconsistent assessment results and can be regarded as a measure of homogeneity of the image assessment [10]. Acceptable values for good alpha coefficients are 0.7 and above [11, 12]. The alpha coefficients in this study were 0.981 for the assessment of age, 0.890 for BMI, 0.892 for facial attractiveness, 0.895 for facial unattractiveness, 0.607 for temporal hollowing, 0.628 for lower cheek fullness, 0.859 for jawline contour, and 0.906 for nasolabial fold severity. These values indicate that despite the large number (27) of ethnically diverse raters, the internal consistency across the assessed scores was very high supporting the validity of the presented results.

The results revealed overall that the change in light angle from 0° to 30° and to 60° influenced total facial assessment and more than regional facial scores. This is indicated by the low alpha coefficient values and by the inconsistent results (Table 1) of the assessment of the temple, the cheeks, the jawline, and the nasolabial sulcus when compared to the parameters from the assessment of facial attractiveness, BMI or age. Facial attractiveness decreased, facial unattractiveness increased, and the evaluated BMI (based on facial assessment) increased statistically significantly at 60°. No such statistically meaningful changes were observed at 30° except for BMI. The explanation for the significant results observed for the BMI assessment is the classification of the (linear) BMI values into categories (1: < 25 kg/m^2^; 2: 25–30 kg/m^2^; 3: > 30 kg/m^2^); this increases the power but decreases the accuracy and could explain the statistically significant results at 30°.

None of the regional scores reached statistical significance at 30° light angle and only the assessment of the nasolabial sulcus reached statistical significance at 60°. The nasolabial sulcus is the result of the descent of the superficial nasolabial fat compartment during aging [13, 14] which prolapses superficial to the sulcus. The severity of the sulcus depends on the light and shadow relationship at the sulcus with more shadow resembling a deeper and thus more severe sulcus. The results are in line with this clinical observation and it seems plausible that with increasing light angle the shadow-component of the sulcus increases whereas the light-component decreases. The results, however, indicate that at 30° of light angle no statistically significant changes are detectable when assessing the nasolabial sulcus for its severity.

Extrapolating the study results it seems that the influence of the light angle is a threshold determined influence rather than a gradual one. This could be based on the fact that at 30° light angle none (except the assessment of the BMI) of the assessed variables changed on a statistically significant level. Statistically meaningful changes were observed only at 60° light angle which include the nasolabial sulcus as a regional facial assessment. Clinically, this would indicate that alterations in the angle of light up to 30° would not influence the perception of a patient’s face on a statistically meaningful level. Additionally, this would indicate that there is an observer blind range (0°–30°) in light angle, in which changes between a baseline and a follow-up image can be captured without having to expect a meaningful change in facial perception. However, it should be mentioned that this observer blind range is only valid if all other imaging parameters are kept constant and the only influencing factor is the change in light angle. Furthermore, it is unclear at what angle when passing the 30° threshold a meaningful change occurs, i.e., at 35° or at 55° for instance. This will be subject to further investigation.

Another interesting finding of this study is that the assessed regional facial scores, i.e., temporal hollowing, lower cheek fullness, and jawline contour showed no statistically meaningful changes both at 30° and at 60° light angle. This is an interesting finding as it could have been expected that the regional scores would be affected on a greater level than the total facial scores. It could be speculated that the assessment of the regional scores is based on a proportionate approach, i.e., the distances and relationship to other facial features which was not influenced by the change in light angle. As these proportions did not change, it may plausibly explain why no statistically meaningful changes were observed. In contrast, total facial assessment and total facial perception seem to be based on light and shadow reflections (and less on proportions) which could account for the results presented herein. Changes in light angle above the identified 30° threshold seem to influence the perception of facial attractiveness and BMI statistically significantly. These results indicate that aesthetic practitioners should be mindful of the significant impact of altering lighting angle when capturing and presenting their treatment results, as the subjective perception of images may change significantly.

## Conclusion

The results of this randomized and blinded investigation revealed that alterations in the angle of light during 2D facial imaging can influence the perception of the face. Facial attractiveness decreased, facial unattractiveness increased, and the evaluated BMI (based on facial assessment) increased statistically significantly at 60°. Regional facial scores, i.e., temporal hollowing, lower cheek fullness, and jawline contour, showed no statistically meaningful changes both at 30° and at 60° light angle. The results indicate that there might be an observed blind range in light angle (0°–30°) which does not influence facial assessment. Increasing the light angle past the threshold value to 60° might statistically significantly influence facial perception which should be accounted for when documenting and/or presenting facial 2D images.
